# Anterograde trans-neuronal labeling of striatal interneurons in relation to dopamine neurons in the substantia nigra pars compacta

**DOI:** 10.3389/fnana.2024.1325368

**Published:** 2024-02-28

**Authors:** Fuyuki Karube, Yang Yang, Kenta Kobayashi, Fumino Fujiyama

**Affiliations:** ^1^Laboratory of Cytology and Histology, Faculty of Medicine, Hokkaido University, Sapporo, Japan; ^2^Section of Viral Vector Development, National Institute for Physiological Sciences, Okazaki, Japan

**Keywords:** substantia nigra, striatum, dopamine, neural tracing, trans-neuronal

## Abstract

Recent advances in neural tracing have unveiled numerous neural circuits characterized by brain region and cell type specificity, illuminating the underpinnings of specific functions and behaviors. Dopaminergic (DA) neurons in the midbrain are highly heterogeneous in terms of gene and protein expression and axonal projections. Different cell types within the substantia nigra pars compacta (SNc) tend to project to the striatum in a cell-type-dependent manner characterized by specific topography. Given the wide and dense distribution of DA axons, coupled with a combination of synaptic and volume transmission, it remains unclear how DA release is spatially and temporally regulated, to appropriately achieve specific behaviors and functions. Our hypothesis posits that hidden rules governing synapse formation between pre-synaptic DA neuron types and striatal neuron types may modulate the effect of DA at a single-cell level. To address this conjecture, we employed adeno-associated virus serotype 1 (AAV1) to visualize the neural circuitry of DA neurons. AAV1 has emerged as a potent anatomical instrument capable of labeling and visualizing pre- and post-synaptic neurons simultaneously through anterograde trans-synaptic labeling. First, AAV1-Cre was injected into the SNc, resulting in Cre expression in both medium spiny neurons and interneurons in the striatum. Due to the potential occurrence of the retrograde transfer of AAV1, only striatal interneurons were considered for trans-synaptic or trans-neuronal labeling. Interneuron types expressing parvalbumin, choline acetyltransferase, somatostatin, or nitrogen oxide synthase exhibited Cre expression. Using a combination of AAV1-Cre and Cre-driven fluorophore expressing AAVs, striatal interneurons and the axons originating from the SNc were visualized in distinct colors. Using immunofluorescence against neurotransmitter transporters, almost all axons in the striatum visualized using this approach were confirmed to be dopaminergic. Moreover, individual DA axons established multiple appositions on the somata and proximal dendrites of interneurons. This finding suggests that irrespective of the extensive and widespread axonal arborization of DA neurons, a particular DA neuron may exert a significant influence on specific interneurons. Thus, AAV1-based labeling of the DA system can be a valuable tool to uncover the concealed rules governing these intricate relationships.

## 1 Introduction

Dopaminergic (DA) neurons in the mesencephalon play a pivotal role in value processing, motivation and learning, and decision-making, contributing to the execution of adaptive behaviors. Among the DA nuclei, the substantia nigra pars compacta (SNc) is a key component of the basal ganglia system, and intensively innervates the dorsal striatum (François et al., 1999; for review, see Bjorklund and Dunnett, 2007). Notably, axon collaterals from individual DA neurons are recognized for their remarkable density with numerous varicosities (Gauthier et al., 1999; Prensa and Parent, 2001; Matsuda et al., 2009). In addition, the spatial extent of the axonal territory of a single DA neuron encompasses a vast area within the striatum (Matsuda et al., 2009). The precise timing and spatial extent of DA release and diffusion emerge as crucial factors for the modulation of activities of neurons and synapses in the striatum to adapt and exhibit appropriate plasticity (for review, see Arbuthnott and Wickens, 2007; Liu et al., 2021). Nevertheless, the mechanisms underlying the requisite control of DA amidst such extensive and apparently less selective axonal arborization remain unknown.

Recent methodological advancements have further promoted the identification of neuron types with the aid of transgenic animals and viral vectors, optogenetics, chemogenetics, and simultaneous detection of multiple RNAs at the single cell level, including RNA sequencing. Extensive research in various brain regions consistently reported that neurons releasing fast neurotransmitters such as glutamate and GABA, exhibit a significant correlation between neuron types, connection probability, and characteristics of synaptic transmission. Neuroscience has made substantial progress over the past decades, revealing that the formation and function of synapses are profoundly affected by the differentiation of neuron types (for review, see Luo et al., 2018; Mukamel and Ngai, 2019; Luo, 2021). In the context of the DA system, studies in mice have reported topographical projections of SNc DA neurons correlated to variations in gene expression and/or external inputs (Watabe-Uchida et al., 2012; Menegas et al., 2015, 2018; Poulin et al., 2018; Luppi et al., 2021). These topographic projections can restrict the spatial extent of regions where DA works efficiently. For recipients of DA from the SNc, striatal neurons are composed of two types of projection neurons: direct and indirect pathway medium spiny neurons (dMSNs and iMSNs), along with interneurons. Interneurons are broadly categorized into cholinergic and GABAergic neurons (Kawaguchi, 1993; for review, see Kawaguchi et al., 1995; Tepper et al., 2010). Recent studies have provided detailed insights into GABAergic interneuron types, further subdividing them into complex groups (Ibanez-Sandoval et al., 2011; Munoz-Manchado et al., 2016, 2018; Assous et al., 2018; for review, see Tepper et al., 2018). In general, gene and protein expressions are highly correlated with the physiological and morphological characteristics of interneurons. For example, striatal fast-spiking neurons express parvalbumin (PV) while low-threshold spiking (LTS) neurons frequently express somatostatin (SOM), nitric oxide synthase (NOS), and/or neuropeptide Y (NPY). Most of the LTS neurons express all of the above three proteins; however, SOM, NOS, and NPY are not completely co-expressed (Munoz-Manchado et al., 2016). In addition, neuroglia form cells, which are not LTS neurons, also express NPY (Ibanez-Sandoval et al., 2011). Dopamine receptors are not only expressed in MSNs but also interneurons, depending on their specific types (for review, Smith and Villalba, 2008; Kreitzer, 2009). These receptors influence the activity of interneurons, which, in turn, modulates MSN activities in an interneuron type-specific manner (Straub et al., 2016; for review, Silberberg and Bolam, 2015). Moreover, considering the heterogeneity of striatal interneurons, changes induced by DA can impact striatal circuitry through various neurotransmitters, neuropeptides, and neuromodulators, such as GABA, acetylcholine, and nitric oxide. Recently, Chuhma et al. (2023) reported that the effects of DA varied among the regions of the striatum, as well as neuron types. It remains unclear whether the heterogeneous transmission of DA is related to the types of SNc DA neurons and striatal neurons. We anticipate that unraveling the relationship between DA neuron types and striatal interneuron types will provide new insights into the basal ganglia circuitry.

Moreover, in contrast to fast neurotransmitters, DA release occurs at both synaptic and non-synaptic sites. Electron microscopic observations have revealed that varicosities in DA axons are filled with vesicles containing DA. Approximately 30–40% of DA release sites form synaptic structures, whereas others lack identifiable synaptic structures (Descarries et al., 1996). At synaptic sites, DA is released into the synaptic cleft and binds to DA receptors on postsynaptic structures. In contrast, DA released at non-synaptic sites is involved in what is commonly referred to as volume transmission, where the released DA is likely to lack specificity for target neurons, diffusing widely and potentially binding to receptors located distal to the release site by chance (for review, see Arbuthnott and Wickens, 2007; Liu et al., 2021). In addition, recent studies have shown the presence of molecular machinery at non-synaptic DA release sites. Similar to fast neurotransmission, active zone-like components are necessary for axonal DA release. There has been an ongoing debate regarding whether numerous varicosities on DA axons that lack active zone-like structures can actually release DA (Daniel et al., 2009; Lipton et al., 2018; Liu et al., 2018; for review, see Liu and Kaeser, 2019). It has been reported that approximately 80% of varicosities in DA axons can be silent, meaning they do not release DA, even when action potentials propagate along the axon in slice preparations (Pereira et al., 2016). In addition, the postsynaptic structures, which receives DA terminal innervation, are not uniform. Similar to GABAergic synapses, gephyrine and neuregulin 2 were localized in the postsynaptic structures which are innervated by the DA terminals with active zone-like properties (Uchigashima et al., 2016; Kim et al., 2023). Thus, questions arise regarding the DA system: why do such heterogeneous DA varicosities and release systems exist, and how are they differentiated in a functionally meaningful manner within the dense and complex axons of single DA neurons?

To address the questions raised above, it would be beneficial to simultaneously label SNc DA neurons and the striatal neurons receiving DA. In this study, we aimed to evaluate the feasibility of using an anterograde trans-neuronal viral vector, specifically adeno-associated virus serotype 1 (AAV1), for this purpose (Zingg et al., 2017, 2020; Li et al., 2021). Previous reports have raised valid concerns regarding the suitability of AAV1 labeling for nigrostriatal projections due to their reciprocal connections (Zingg et al., 2020, 2022). To circumvent this issue, we employed AAV1 to investigate DA neurons and the striatal interneurons with their axons confined within the striatum.

## 2 Materials and methods

### 2.1 Animals

All animal experiments were designed and approved by the Animal Care and Use Committee of Hokkaido University (Approval Number: 20–0106). Experiments involving adeno-associated viral (AAV) vectors were approved by the Safety Committee on Genetic Recombination Experiments of Hokkaido University (Approval Number: 2020–019). Fifteen male C57BL/6 J mice (8–12 weeks) were used for the present experiments.

### 2.2 Viral vectors

AAVs were obtained from Addgene (Watertown, MA, United States). For trans-neuronal labeling, the AAV1-hSyn-Cre (Addgene #10553) was used. For Cre-dependent expression of fluorophores, the AAVdj-EF1α-double floxed-hChR2(H134R)-mCherry-WPRE-HGH-pA (Addgene #20297) and/or AAVdj-Syn-FLEX-rc[Chronos-GFP] (Addgene #62722) were used. In the present study, these AAVs were exclusively used only for Cre-dependent expression of fluorescent proteins. Therefore, hereafter we refer to them as AAV-Flex-mCherry and AAV-Flex-GFP, respectively.

### 2.3 Animal surgery

The mouse was anesthetized using a combination anesthetic consisting of three reagents: 0.3 mg/kg of medetomidine (Domitor Injection, Nippon Zenyaku Kogyo Co., Ltd., Koriyama, Japan), 4.0 mg/kg of midazolam (Dormicum Injection, Maruishi Pharmaceutical Co., Ltd., Osaka, Japan), and 5.0 mg/kg of butorphanol (Meiji Seika Pharma Co., Ltd., Tokyo, Japan). Henceforth, this mixture is referred to as MMB, and was administered subcutaneously at a dose of 0.05 mL per 10 g of body weight. Throughout the surgery, the depth of anesthesia was monitored by observing the heart and respiration rates. Additional MMB (0.02 mL for each time) was administered to keep appropriate states. The anesthetized mouse was securely positioned using a stereotaxic device (Narishige, Tokyo, Japan) with ear bars and a nose bar. The body temperature of the mice was continuously monitored and maintained at approximately 38°C (BWT 100A animal warmer; Bio Research Center, Nagoya, Japan).

To prepare for surgery, the fur on the head was trimmed with scissors, and local anesthesia (xylocaine gelée, Sandoz K.K., Tokyo, Japan) was applied to the skin. The skin was incised using scissors to expose the skull. The nose bar was adjusted to align with the heights of the bregma and lambda. Small holes were drilled in the skull using a dental drill to expose the dura mater. The coordinates for injection sites on Mouse Brain Atlas (Paxinos and Franklin, 2013) were [3.1 mm posterior to the bregma (A -3.1), 1.5 mm lateral from the midline (LM 1.5), 3.8 mm deep from the cortical surface (D 3.8)] for injections into the substantia nigra and [A + 0.5, L 2.0, D 2.2] for injections into the striatum.

For retrograde tracer injection in the striatum, a 5% solution of FluoroGold (FG, Fluorochrome, Denver, CO, United States) was prepared in 0.1 M phosphate-buffered saline (PBS, pH 7.4). The solution of AAV or FG was injected using brief pulses of air pressure with a picopump (PV820, World Precision Instruments, Sarasota, FL, United States). A glass needle with a tip diameter of 20 μm, containing the solution, was positioned at the designated coordinates and maintained in place for ~5 min before injection. Subsequently, the solution was injected using air pulses of 5 ms (20 psi) at a rate of ~0.3 Hz. In our setup, it took approximately 10 min to eject 200–500 nL of the solution. Following injection, the glass needle remained *in situ* for 10 min before being slowly withdrawn. Once all injections were completed, the skull and surrounding skin were rinsed with saline, and the skull holes were sealed with bone wax. Finally, the skin was sutured and disinfected using 10% povidone iodine solution. After surgery, the mouse was kept on a heated pad until it fully recovered from anesthesia, and nanoxane was administered subcutaneously to alleviate pain. In the first series of AAV injections (N = 4), AAV1-hSyn-Cre was injected into the SNc of one hemisphere and the mouse was perfused 1 week after injection. In the second series of AAV injections (*N* = 4), AAV1-hSyn-Cre was injected into the SNc of one hemisphere. After 1 week, AAV-Flex-GFP was injected into the striatum of both hemispheres, and AAV-Flex-mCherry was injected into the SNc in the injected hemisphere using the same coordinates as AAV1-hSyn-Cre. The reverse combination of AAV-Flex-mCherry and AAV-Flex-GFP was applied for one of 4 mice. Two weeks later, the mice were perfused as described below. For FG injection (*N* = 4 mice), the mice were perfused 4–5 days after injection.

### 2.4 Histology

#### 2.4.1 Perfusion

The mice were deeply anesthetized with pentobarbital (intraperitoneal injection at a dose of 100 mg/kg, Tokyo Chemical Industry Co., Ltd., Tokyo, Japan). A total of 20 mL of a pre-fixative solution consisting of 50 mM MgCl_2_ and 7.5% sucrose in 0.02 M phosphate buffer (PB) was perfused through the aorta. Subsequently, approximately 50 mL of the fixative solution containing 4% paraformaldehyde (Nacalai Tesque, Tokyo, Japan) and 0.2% picric acid (Nacalai Tesque) in 0.1M PB was perfused. Following a post-fixation period of 2.5 h *in situ*, the brain was removed and placed in 0.1 M PB for rinsing. After three times rinses (each lasting 30 min), the brain was immersed in 15% sucrose in 0.1 M PB until it sank, followed by transfer to 30% sucrose solution in 0.1 M PB. Once the brain had sunk, it was transferred to a new solution containing 0.02% sodium azide, and the brain was stored at 4°C until further use.

#### 2.4.2 Tissue sectioning and immunofluorescence

The brain was sectioned using a freezing microtome (Leica SM2000R, Leica Microsystems, Wetzlar, Germany) in either a coronal or sagittal plane with a thickness of 25 μm for each section. Subsequently, the sections underwent three 10-min washes in 0.1 M PB. For antibody reactions, the sections were immersed in an incubation buffer [comprising 10% normal goat serum, 2% bovine serum albumin, 0.02% sodium azide, and 0.5% Triton X in 0.05 M Tris-buffered saline (TBS)], along with the appropriate concentration of antibodies (Table 1). In the case of the primary antibody reactions, the sections were incubated for 24–48 h at room temperature (RT) or for 3–4 days at 4°C. After three rinses with TBS, the sections were incubated with a mixture of secondary antibodies (Thermo Fisher Scientific or Jackson Immunoresearch, West Grove, PA, United States) for 3 h at RT. Subsequently, the sections underwent three additional TBS rinses, and were mounted on glass slides and dried, embedded with ProlongGold (Thermo Fisher Scientific), and cover-slipped. These slides were stored in the dark at 4°C. Immunofluorescence against parvalbumin (PV) and calbindin D-28 k (CB) was used to identify brain regions.

**Table 1:** List of the primary antibodies.

Antigen	Company/catalog ID	Animals	Dilution	RRID
Calbindin	Swant/CB9	Rabbit	4,000	AB_10000348
Choline acetyltransferase	Millipore/AB144P	Goat	500	AB_2079751
Choline acetyltransferase	Millipore/MAB305	Mouse	1,000	AB_94647
High-affinity choline transporter	Frontier Institute/CHT1-Go	Goat	250	AB_2571677
Cre	Millipore/MAB3120	Mouse	500	AB_2085748
Ctip2	Abcam/ab18465	Rat	500	AB_2064130
Dopamine receptor type 2	Frontier Institute/D2R-GP-Af500	Guinea pig	200	AB_2571597
Dopamine transporter	Frontier Institute/DAT-Go-Af980	Goat	1,000	AB_2571687
GFP	GeneTex/13,970	Chicken	1,000	AB_371416
mCherry	Thermo Fisher Scientific/M11217	Rat	1,000	AB_2536611
Nitric oxide synthase	Sigma/N7280	Rabbit	5,000	AB_260796
Neuropeptide Y	Immunostar/22,940	Rabbit	2,000	AB_2307354
Parvalbumin	Swant/PV25	Rabbit	5,000	AB_10000344
Parvalbumin	Synaptic Systems/195,004	Guinea pig	5,000	AB_2156476
Somatostatin	Millipore/MAB354	Rat	250	AB_2255365
Vesicular glutamate transporter 2	Frontier Institute/VGluT2-GP-Af810	Guinea pig	500	AB_2571621
Vesicular GABA transporter	Synaptic Systems/131,003	Rabbit	250	AB_887869

#### 2.4.3 Microscopic observation and morphological analysis

The specimens were observed using two types of fluorescent microscopes: a box-type one (BZ-X710; Keyence, Osaka, Japan), and an epifluorescent microscope (BX51, Olympus, Tokyo, Japan) equipped with a CCD camera (DP73, Olympus) and a CMOS camera (Orca Spark, Hamamatsu photonics, Hamamatsu, Japan). Images were captured using ×4, ×10, and ×20 objectives. For co-expression analysis, a confocal laser microscope (FV1200, Olympus) was utilized to capture higher magnification images using ×40 or oil-immersion objectives (×60 and ×100). The acquired images underwent adjustments for level compensation and were analyzed using Fiji (a branch of ImageJ; Schindelin et al., 2012). For cell counting, Z-stack images of the 3-μm focus step were acquired using FV1200. Neurons were counted in a manner of stereology using Fiji. For the analysis of the co-localization of the AAV labeled axons and dopamine transporter (DAT) or High-affinity choline transporter (CHT1), Z-stack images of 0.3-μm step were acquired by FV1200 using a × 60 objective with ×4 zoom. The resulting voxel size was 0.082 μm × 0.082 μm × 0.3 μm and the size of the individual images was 52.48 μm × 52.48 μm in XY dimensions. Since the immunofluorescence of DAT and CHT1 visualize not only axonal varicosities but also inter-varicose axons, the colocalization was analyzed at the level of the axons, not terminals. Namely, the AAV-labeled axons were traced in a Z-stack image, referred to as axon segments. Subsequently, individual axon segments were investigated for DAT or CHT1 expression. The proportion of DAT or CHT1-expressing axon segments was reported. For the colocalization of the AAV-labeled axons and vesicular glutamate transporter 2 (VGluT2) or vesicular GABA transporter (VGAT), since these proteins are highly localized within axonal varicosities, the colocalization was analyzed at the level of varicosities. The proportion of VGluT2 or VGAT-expressing varicosities was reported. As described in Results 3.1, a small portion of AAV1-labeled varicosities appear to co-localize with immunofluorescence against VGluT2 or VGAT. In some cases, co-localization did not happen in a whole varicosity, suggesting that it may be not co-localization. However, the possible best resolution of our imaging system could not exclude the possibility of co-localization; thus, we considered them as colocalization in this study.

Morphological details of the cell body and dendrites were reconstructed from the Z-stack images using Neurolucida 360 (MBF Bioscience, VT, United States). Putative appositions (PAPs) are defined as the varicosities that appose on the surface of the cell body and proximal dendrites in 3D. PAPs were manually detected.

### 2.5 Statistical analysis

Statistical analysis was carried out using Microsoft Excel, R (R Project for Statistical Computing, Vienna, Austria),^1^ and IgorPro (Wave Metrics Inc., Portland, OR, United States). The averaged values are presented as mean ± SD. Statistical significance for comparing the proportion of Cre-expressing neurons among different neuron types was assessed using Fisher’s exact test with multiple comparison correction using Holme’s method. Comparison of data values among multiple groups was performed using one-way ANOVA with post-hoc Tukey test. *p*-values less than 0.05 were considered statistically significant.

## 3 Results

### 3.1 Cre expression in the striatum was induced by AAV1 injection into the SNc

**Figure 1:**
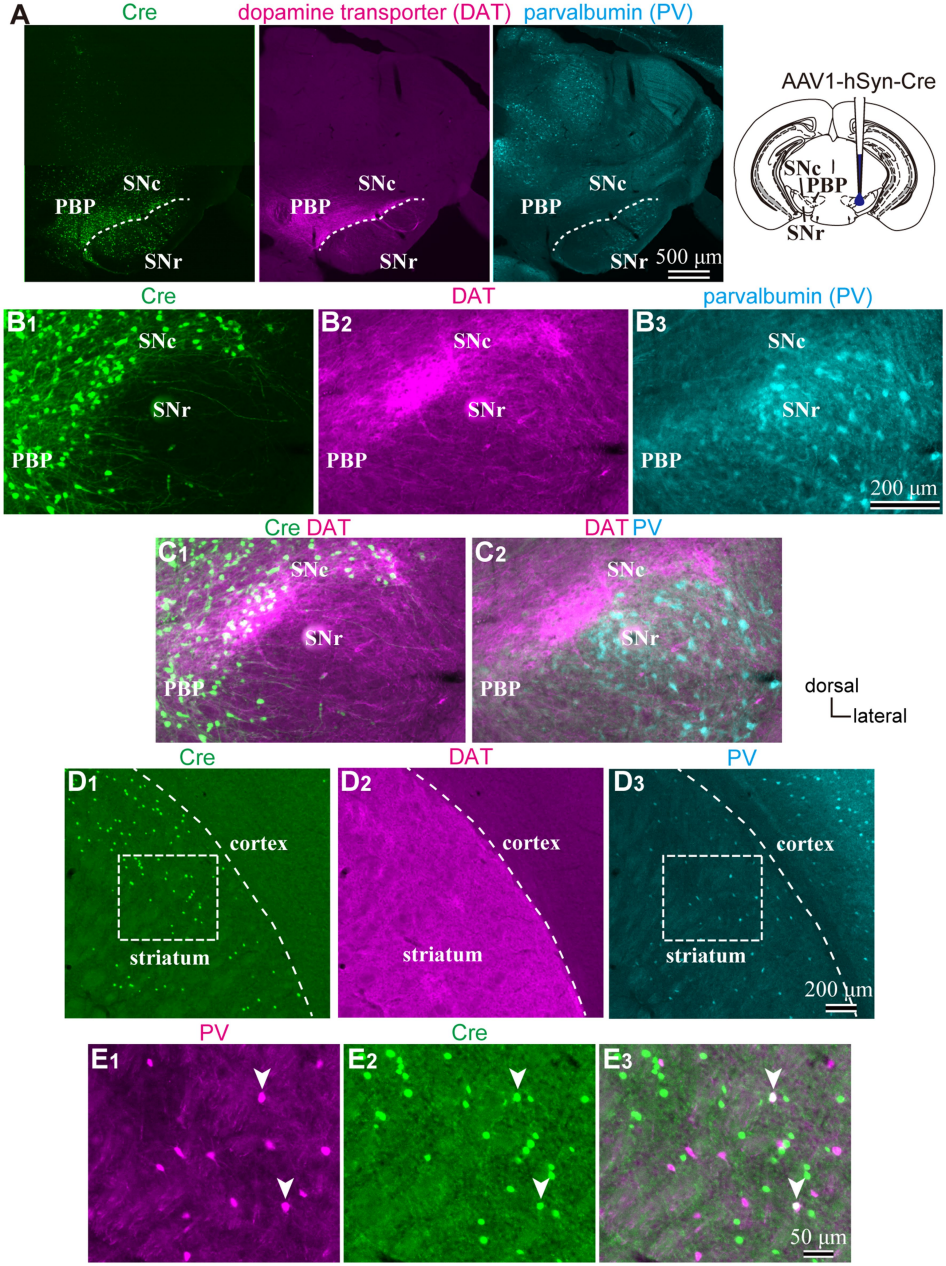
**(A)** An injection site of AAV1-hSyn-Cre to substantia nigra pars compacta (SNc). Immunostaining images against Cre (Left|), dopamine transporter (DAT, middle), and parvalbumin (PV, Left) are represented. Note that Cre is expressed in the SN and more medial parts of the midbrain. **(B)** Magnified images around SN. **(C)** Merged images of those shown in **(B)**. Cre expression was found both in DAT-positive and negative neurons, whereas only a few cells were found in the SN reticulata (SNr). **(D)** Expression of Cre (D1), DAT (D2), and PV (D3) in the striatum derived from the same mouse as shown in **(A–C)**. Cre-expressing neurons scatter in the dorsolateral striatum. **(E)** Magnified views of the rectangle area shown in **(D)**. Note that Cre is expressed in PV-expressing neurons (arrowheads). PBP, parabrachial pigmented nucleus.

**Figure 2:**
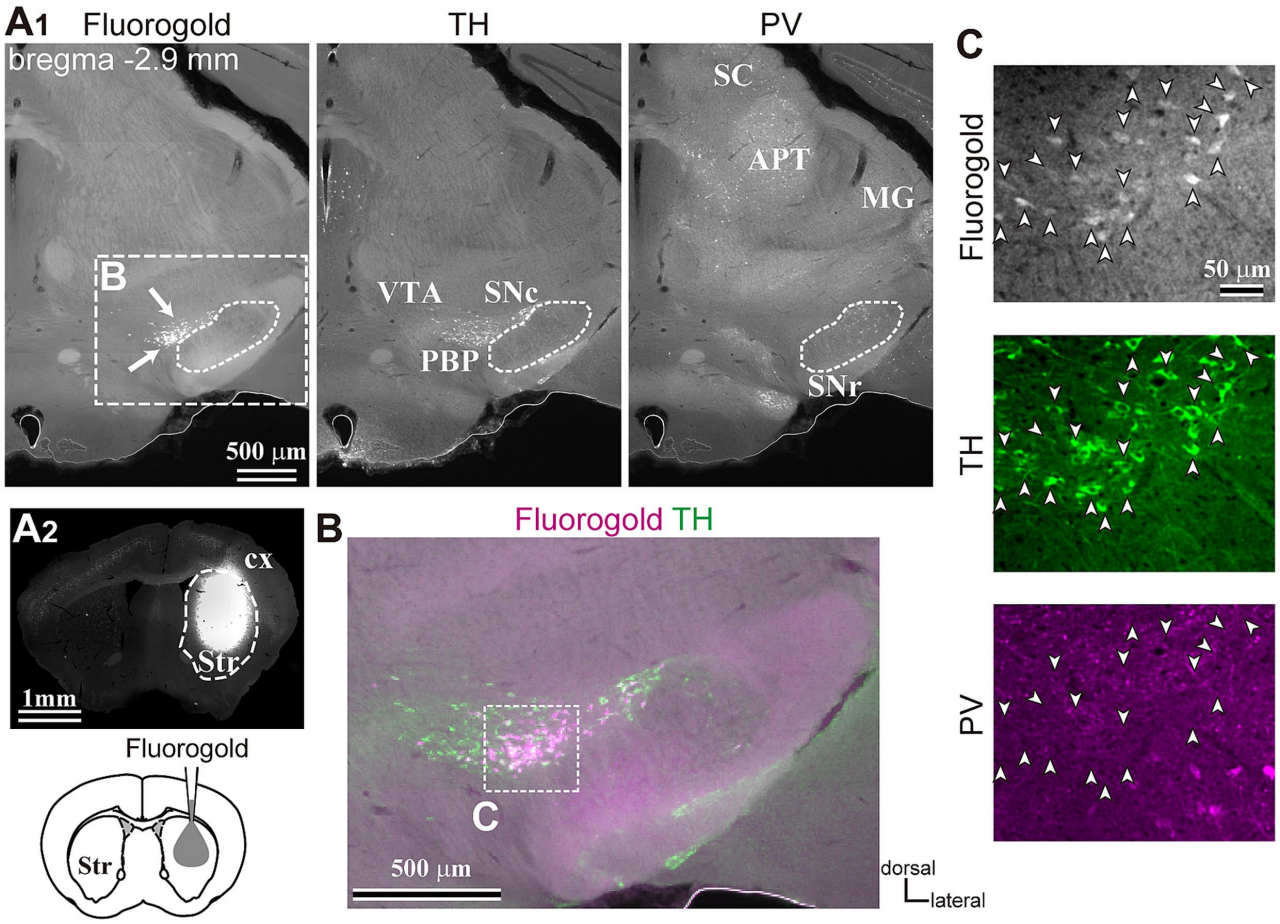
Inputs to the dorsolateral striatum. **(A)** Retrograde tracer (FG) injection in the striatum **(Str, A2)** allowed the visualization of retrogradely-labeled projection neurons in SN (arrows, the left panel in **A1**). The section was immunostained against tyrosine hydroxylase (TH, middle) and parvalbumin (PV, right). Note that only a few cells were labeled apart from those in SN in the midbrain. **(B)** A magnified merged view of the rectangle area shown in **(A1)**. FG is shown in magenta and TH in green. **(C)** Confocal images of the rectangle area shown in **(B)**. Note that all FG labeled neurons (arrowheads, top) co-expressed TH (green, middle), but not PV (magenta, bottom). APT, anterior pretectal nucleus; cx, cerebral cortex; MG, medial geniculate nucleus; PBP, parabrachial pigmented nucleus; SC, superior colliculus; SNc, substantia nigra pars compacta; SNr, substantia nigra pars reticulata; VTA, ventral tegmental area.

**Figure 3:**
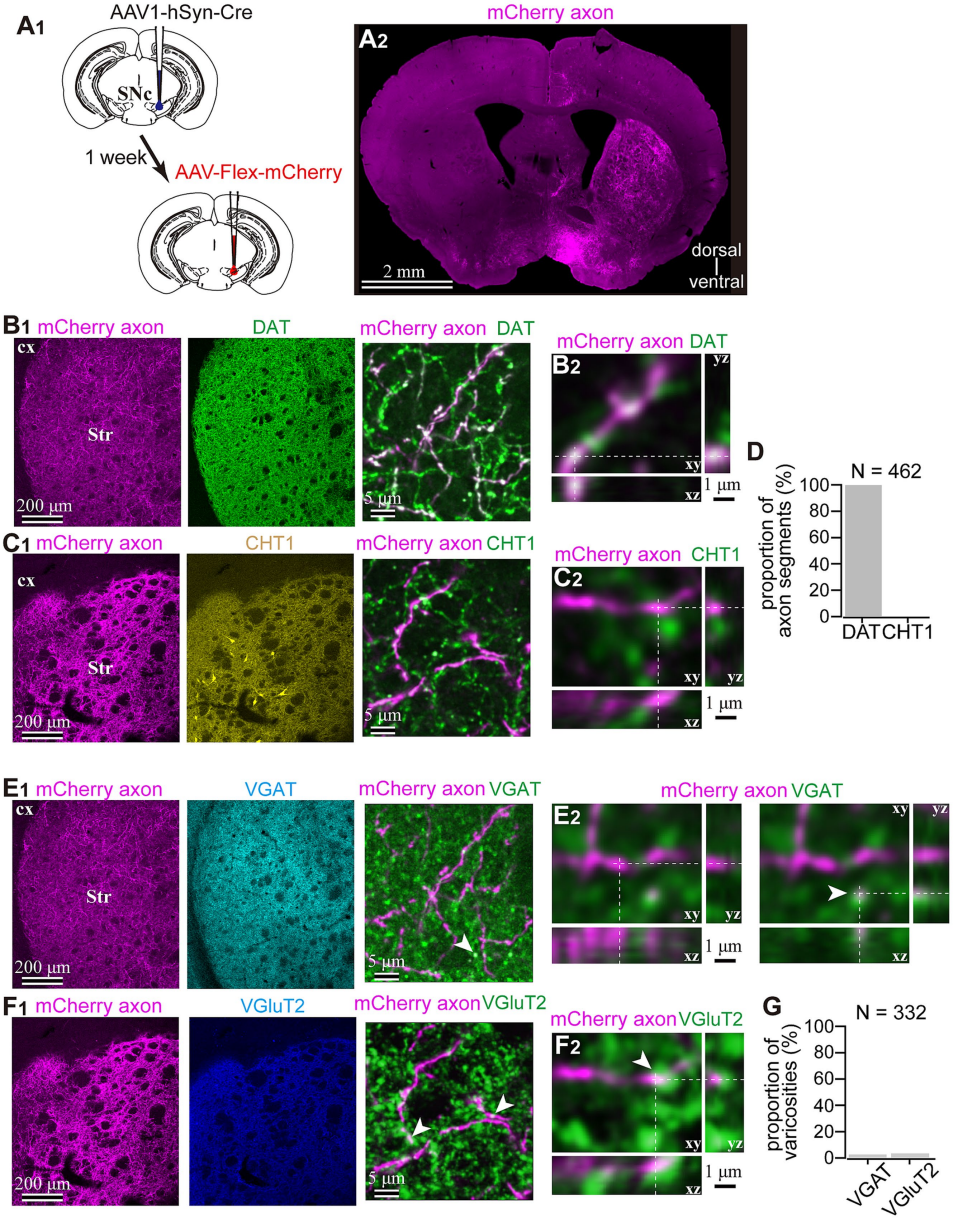
Dopaminergic identity of axons in the striatum labeled by means of the AAV1-Cre injection into the SNc. **(A)** AAV1-dependent labeling of the midbrain neurons. **(A1)** AAV1-hSyn-Cre was injected into the SNc in one hemisphere. After a week, AAV-Flex-mCherry was injected into the SNc at the same coordinates. **(A2)** mCherry labeled axons were found in the striatum ipsilateral to the injection. **(B1,C1)** Characterization of the labeled axons (magenta in **B,C**) in the striatum (Str). Immunostaining against dopamine transporter (DAT; green in **B1**), choline transporter 1 (CHT1; yellow in the middle panel of **(C1)**, in the right panel, shown in green to distinguish co-localization clearly). **(B2,C2)** Confocal images with higher magnification are presented in xy, xz, and yz planes. Note the colocalization of DAT in mCherry axons **(B2)**, whereas there was no colocalization of CHT1 in mCherry axons. **(D)** Quantification of colocalization of DAT or CHT1. **(E1,F1)** Epifluorescent immunofluorescence against vesicular GABA transporter (VGAT, cyan in **E1** middle panel and green in **E1** right panel) and vesicular glutamate transporter 2 (VGluT2, blue in **F1** middle panel and green in **F1** right panel). **(E2,F2)** Confocal images with higher magnification are presented in xy, xz, and yz planes. In most of the mCherry axon terminals, VGAT or VGluT2 expression was not observed. However, occasionally VGAT (arrowhead in **E2**) or VGluT2 (arrowhead in **F2**) was overlapped with mChery axons, while the shape of the terminals was apparently not identical. **(G)** Proportion of AAV1-labeled varicosities with immunofluorescence against VGluT2 or VGAT.

To reveal the applicability of anterograde-trans-neuronal AAV1 in DA neuronal circuits, AAV1-hSyn Cre was injected into the SNc of one hemisphere in wild-type mice (Figure 1). One week later, the mice were perfused to examine the expression of Cre in the SNc and the striatum using immunofluorescence (*N* = 4 mice). As a result, we confirmed the presence of cell bodies with Cre expression in their nuclei within the injected area (Figure 1A). Given the difficulty in precisely restricting AAV1 injection to the SNc alone, both DA and non-DA neurons expressed Cre, as revealed by triple immunostaining (Figures 1A–C). We also observed Cre expression in the striatum on the same hemisphere where the AAV1-Cre was injected (Figure 1D). Cell bodies with Cre expression were scattered throughout the striatum and some of the Cre-expressing neurons co-expressed parvalbumin (PV) (Figure 1E). As previously mentioned, AAV1-Cre infected not only DA neurons but also other types of neurons in the midbrain near the SNc. Consequently, it was unclear whether striatal Cre expression originated from DA neurons. To confirm this, we initially visualized neurons projecting to the striatum from the whole brain using a retrograde tracer, FluoroGold (FG), injected into the dorsolateral striatum (*N* = 4 mice; Figure 2A). The result showed that only a few non-DA neurons in the dorsal midbrain were retrogradely labeled (Figure 2A; Supplementary Figure S1). The result suggests that even if the leak of tracers happened along a glass needle track toward the SN, the labeling derived from other than the SN can be neglectable. In SN, the proportion of DA neurons to the total FG labeled neurons was 95.0 ± 1.1% (*N* = 733 FG labeled neurons; *N* = 4 mice; *N* = 2 sections per mouse; Figures 2B,C). Only 4 PV-expressing neurons among 733 FG neurons were observed in the SN. Therefore, the SN DA neurons projecting to the striatum can be reliably labeled by AAV injection to the SN without the aid of transgenic animals. Next, we combined AAV1-Cre injection with a subsequent AAV-Flex-mCherry injection at the same coordinates within the SNc. As shown in Figure 3A, we did not observe fluorescence in the contralateral striatum to AAV1-Cre injected SNc, whereas we detected bright mCherry signals in the ipsilateral hemisphere where the injection into the SNc was performed, indicating negligible level of mCherry expression without Cre. To clarify the identity of the mCherry-labeled axons in the striatum, we examined the neurotransmitter transporters expressed in them using immunostaining against dopamine transporter (DAT), high-affinity choline transporter (CHT1), vesicular GABA transporter (VGAT), and vesicular glutamate transporter 2 (VGluT2). These four transporters are known to be expressed in the midbrain neurons. If the labeled axons expressed DAT, they could be considered axons from DA neurons. The results revealed that all labeled axon segments from the ventral midbrain (labeled by mCherry in Figure 3) expressed DAT (Figure 3B), while none of them expressed CHT1 (Figure 3C), strongly indicating their dopaminergic nature and the absence of contamination with axons from cholinergic neurons (Figure 3D; DAT expression in 462/462 axon segments; CHT1 in 0/462, *N* = 4 mice). Conversely, we observed VGAT or VGluT2 expression in only a very small number of varicosities among the varicosities of the labeled axons (Figures 3E–G; *N* = 4/138 for VGAT; *N* = 14/388 for VGluT2; *N* = 4 mice). In all 18 axon segments containing the varicosities with VGAT or VGluT2 expression, it is not the case that all the varicosities in the corresponding axon segments expressed VGAT or VGluT2. They were sporadically distributed instead, suggesting that these axons are not purely glutamatergic or GABAergic.

**Figure 4:**
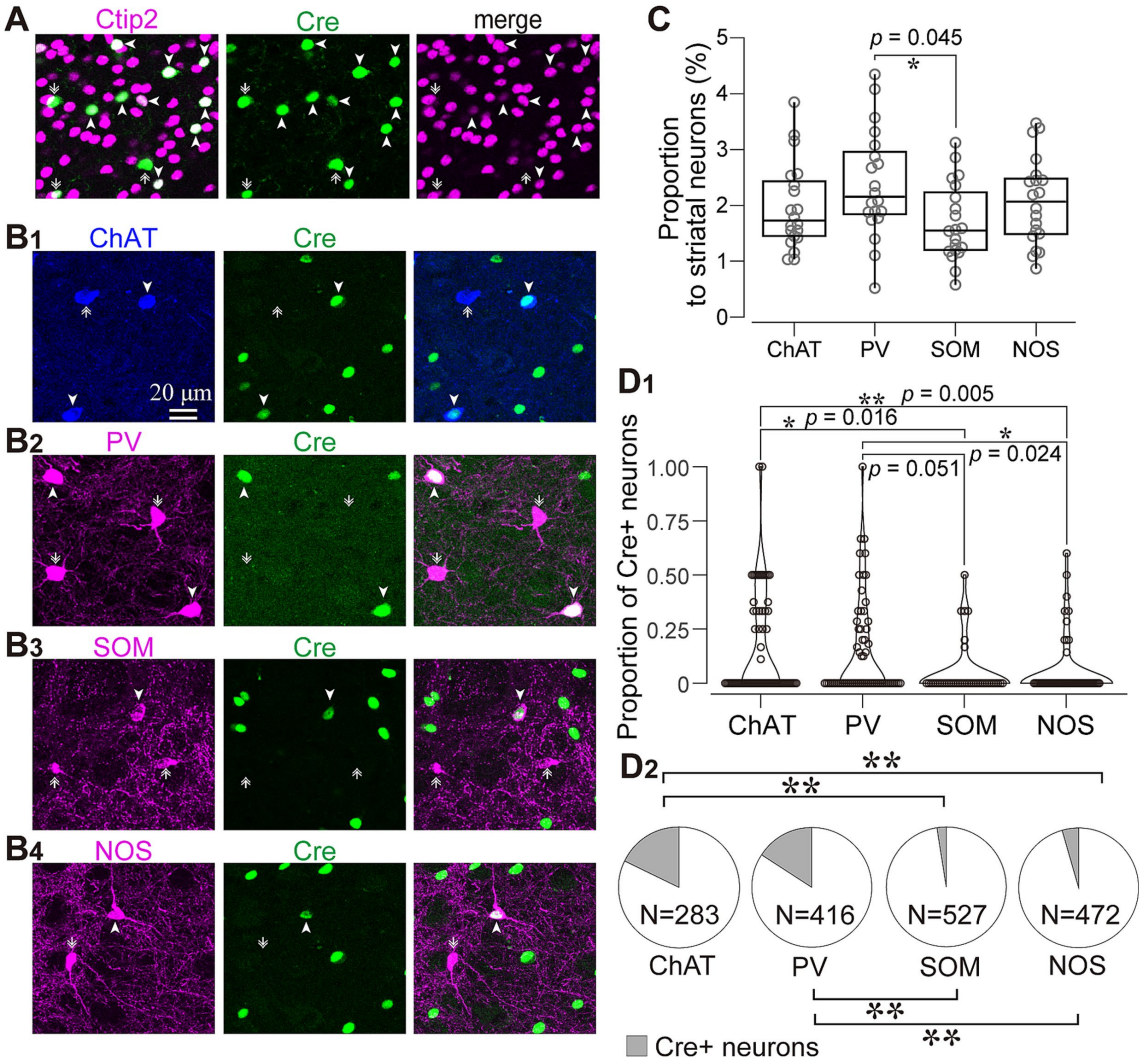
Immunofluorescent identification of striatal cell types visualized by AAV1 labeling. **(A)** Ctip2 expression (magenta) in a part of Cre-expressing neurons (green), indicating that they are medium spiny neurons. **(B)** Expression of striatal interneuron markers in Cre-expressing neurons. Images for expression of choline acetyltransferase (ChAT; blue in **B1**), parvalbumin (PV; magenta in **B2**), somatostatin (SOM; magenta in **B3**), and nitric oxide synthase (NOS; magenta in **B4**). The arrowheads denote neurons expressing both Cre and an interneuron marker protein. The arrows identify neurons expressing either of interneuron markers without Cre expression. **(C)** Proportion of four types of interneurons to all NeuN-expressing cells in the intact mice **(D)** Proportion of Cre-expressing neurons for each interneuron type. See text and Table 3 for details. **(D1)** Proportion of Cre-expression neurons to the number of each interneuron type in each ROI. **(D2)** Proportion of Cre-expressing neurons as the sum of all ROIs. ChAT and PV positive neurons more frequently expressed Cre.

As the aforementioned results indicated that almost all axons from AAV1-labeled neurons were dopaminergic, we examined which types of striatal neurons expressed Cre. To address this question, immunofluorescence targeting striatal neuron markers was combined with Cre immunostaining. We observed that a subset of neurons expressing Cre also co-expressed Ctip2, a marker of MSNs (Arlotta et al., 2008; Figure 4A). Since AAV1 can be transported both retrogradely and anterograde trans-synaptically (Zingg et al., 2017, 2020), it is challenging to differentiate between anterograde trans-neuronal labeling and retrogradely transported labeling. Therefore, we excluded MSNs from further analyses in this study. However, to characterize the labeling of interneurons, some indices were also quantified in MSNs for comparison. In contrast, since the axons of interneurons are confined to the local striatal area and do not extend beyond, Cre expression in interneurons can be considered as anterograde trans-neuronal labeling. Indeed, we detected Cre-expression in interneurons (Figure 1E). To determine the cell types of interneurons, we examined the expression of four representative interneuron markers, choline acetyltransferase (ChAT), PV, somatostatin (SOM), and nitric oxide synthase (NOS) (Figure 4B).

We wondered if there was any potential preference in Cre expression among different interneuron types. To investigate this, we quantified the proportion of neurons expressing ChAT, PV, SOM, and NOS in the dorsolateral striatum using a combination with immunolabeling against NeuN in intact mice (*N* = 20 sections from 3 mice without AAV injection). While the proportion of PV-expressing neurons was slightly higher than that of SOM-expressing neurons, the proportion of PV, ChAT, SOM, and NOS were relatively similar to each other (Figure 4C; PV, 2.46 ± 0.35%; ChAT, 1.94 ± 0.50%; SOM, 1.72 ± 0.07%; NOS, 2.09 ± 0.12%, *p* = 0.045 for PV and SOM as determined by post-hoc Tukey test), consistent with a previous report (Munoz-Manchado et al., 2016). The number of neurons counted was summarized in Table 2.

**Table 2:** Total counts of the striatal interneurons in intact mice.

Interneuron marker	Number of immunopositive neurons	Number of neurons	Proportion of each interneuron (%)
ChAT	124	6,210	2.00
PV	147	6,467	2.27
NOS	130	6,308	2.06
SOM	105	6,308	1.66

Next, we quantified the proportion of AAV1-driven Cre expression in each interneuron type within the region of interest (ROI; *N* = 4 mice). These ROIs were established in the dorsolateral striatum, where Cre expression was observed (ROIs, 318 μm × 318 μm for each ROI; see Table 3 for details). If a given ROI did not contain Cre expression or any neuron with a particular interneuron marker, we excluded it from the count for that marker. As expected from the low total proportion of interneurons within the striatal neuron population (Graveland and Difiglia, 1985), the number of Cre-expressing interneurons was smaller than that of MSNs. Among interneurons, the proportion was significantly higher for ChAT and PV neurons compared to the other interneuron types (Figure 4D1; Table 3). In addition, the coefficient of variation (CV) values were larger in ChAT, PV, and NOS-expressing neurons. The proportion of the total number of Cre-expressing neurons to the number of neurons expressing each marker was calculated, as the sum of all ROIs (Figure 4D2). This proportion was 17.7% for ChAT (*N* = 283 ChAT neurons), 15.6% for PV (*N* = 416), 4.2% for NOS (*N* = 472), 2.5% for SOM (*N* = 527), and 8.1% for Ctip2 (*N* = 14,200). Once again, the value was significantly higher in ChAT and PV neurons compared to SOM or NOS neurons, as determined by Fisher’s exact test with value of *p* compensation using Holm’s method (*p* = 7.12 × 10^−4^ for ChAT vs. PV; *p* = 3.37 × 10^−21^ for ChAT vs. SOM; *p* = 4.58 × 10^−16^ for ChAT vs. NOS; *p* = 5.58 × 10^−13^ for PV vs. SOM; *p* = 2.58 × 10^−8^ for PV vs. NOS; *p* = 0.155 for NOS vs. SOM). The proportion of Cre-expressing neurons in total Ctip2-expressing neurons (1,058 Cre-expressing neurons/14,200 Ctip2-expressing neurons) was slightly smaller than that in the sum of four types of interneurons (148 Cre-expressing neurons/1439 interneurons) (*p* = 0.00022 by Fisher’s exact test), although the comparison may be affected by large differences of the number of samples. To compare the mean and variance of the whole population of interneurons and MSNs, the ROI data from ChAT, PV, NOS, and SOM neurons are pooled. The mean proportion was 8.4 ± 17.8% for total interneurons (395 ROIs) and 7.8 ± 3.9% for Ctip2-neurons (31 ROIs). Although the average values were similar, there is a significant difference due to a large variance for interneurons (*p* = 3.15 × 10^−6^ using Wilcoxon’s signed-rank sum test).

**Table 3:** Cre-expression in each neuron type.

Neuron type marker	Number of ROIs	Proportion of Cre-coexpression in each neuron type in each ROI (%)	CV
ChAT	80	17.4 ± 25.6	1.47
PV	90	14.0 ± 20.3	1.45
NOS	92	4.1 ± 11.6	2.83
SOM	133	2.2 ± 0.8	0.36
Ctip2	31	7.8 ± 3.9	0.5

### 3.2 AAV1-Cre dependent visualization of striatal neurons

**Figure 5:**
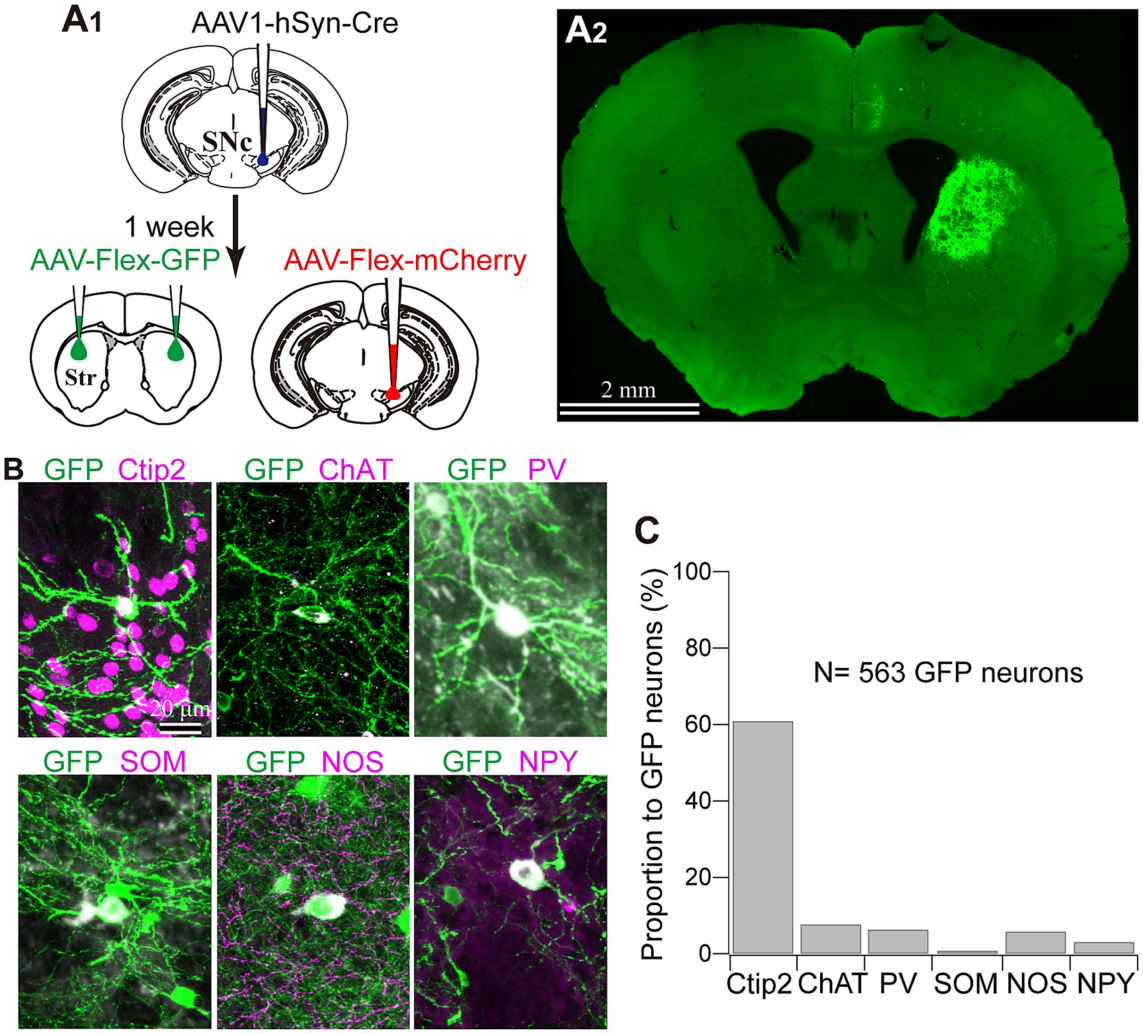
Striatal neuron types labeled with AAV1. **(A)** AAV1-Cre-dependent visualization of striatal neurons. AAVs expressing mCherry and GFP were injected into the SNc and the striatum (Str), respectively, 1 week after AAV1-hSyn-Cre injection to the SNc **(A1)**. Note that GFP expression in the striatum was observed only ipsilaterally to the injected the SNc **(A2)**. **(B)** Representative images of GFP-expressing striatal neurons which co-express either cell type marker proteins. For further details of the marker proteins, see Figure 4 and text. **(C)** Proportion of neurons expressing each cell type marker.

To further confirm whether this transportation was associated with DA axons, we visualized Cre-expressing neurons in both the SNc and striatum. To prove this, 1 week after injecting AAV1-Cre into the SNc of one hemisphere, we injected AAV-Flex-mCherry into the SNc at the same coordinates where AAV1-Cre has been administered. Additionally, we injected AAV-Flex-GFP into the striatum of both hemispheres. We also examined the reverse combination of the above AAVs. As illustrated in Figures 3A and 5A, we observed no fluorescence in the contralateral striatum to the AAV1-Cre injected SNc. In contrast, we detected bright mCherry and GFP signals in the ipsilateral hemisphere where the injection into the SNc occurred. This suggests minimal (if any) leakage in the expression of Cre-driven fluorophores.

Next, we identified the cell types of striatal interneurons labeled by Cre-driven fluorophores expression. Figure 5B shows the somata and proximal dendrites of fluorescently labeled striatal neurons, along with immunofluorescence staining for neuron type markers (*N* = 4 mice; *N* = 433 neurons with triple immunostaining for PV, NOS, and Ctip2; *N* = 130 labeled neurons with triple immunostaining for ChAT, SOM, and NPY). All the markers examined here were detected in fluorescently labeled neurons in an AAV1-Cre-dependent manner. Among them, 60.89% of neurons could be categorized as MSNs based on their Ctip2 expression (Figures 5B,C). ChAT-expressing neurons constituted 7.69% of the total labeled neurons, and this proportion was similar to that of PV (6.35%) or NOS (5.85%) neurons. In the first combination of triple staining, no coexpression was observed among Ctip2, PV, and NOS. In the second combination of triple staining, while NPY and SOM were known to co-express to a considerable extent (Kawaguchi et al., 1995; Munoz-Manchado et al., 2016), the proportion of SOM neurons was relatively smaller (0.77%) compared to that of NPY neurons (3.08%). The remaining proportion of the labeled neurons (approximately 18%) is likely to represent other types of interneurons, primarily due to their aspiny dendrites. When comparing the average proportion of each interneuron type to the total number of neurons in the dorsolateral striatum of the intact mice (Figure 4C; Table 2) with these proportions, it becomes evident that the proportion of AAV1-driven labeled neurons was higher in ChAT (*p* = 5.74 × 10^−11^), PV (*p* = 4.48 × 10^−6^), and NOS neurons (*p* = 6.44 × 10^−6^), while the proportion in SOM neurons was not significantly different (*p* = 0.249) by Fisher’s exact test.

### 3.3 Relationship between single dopamine axons and striatal interneurons

**Figure 6:**
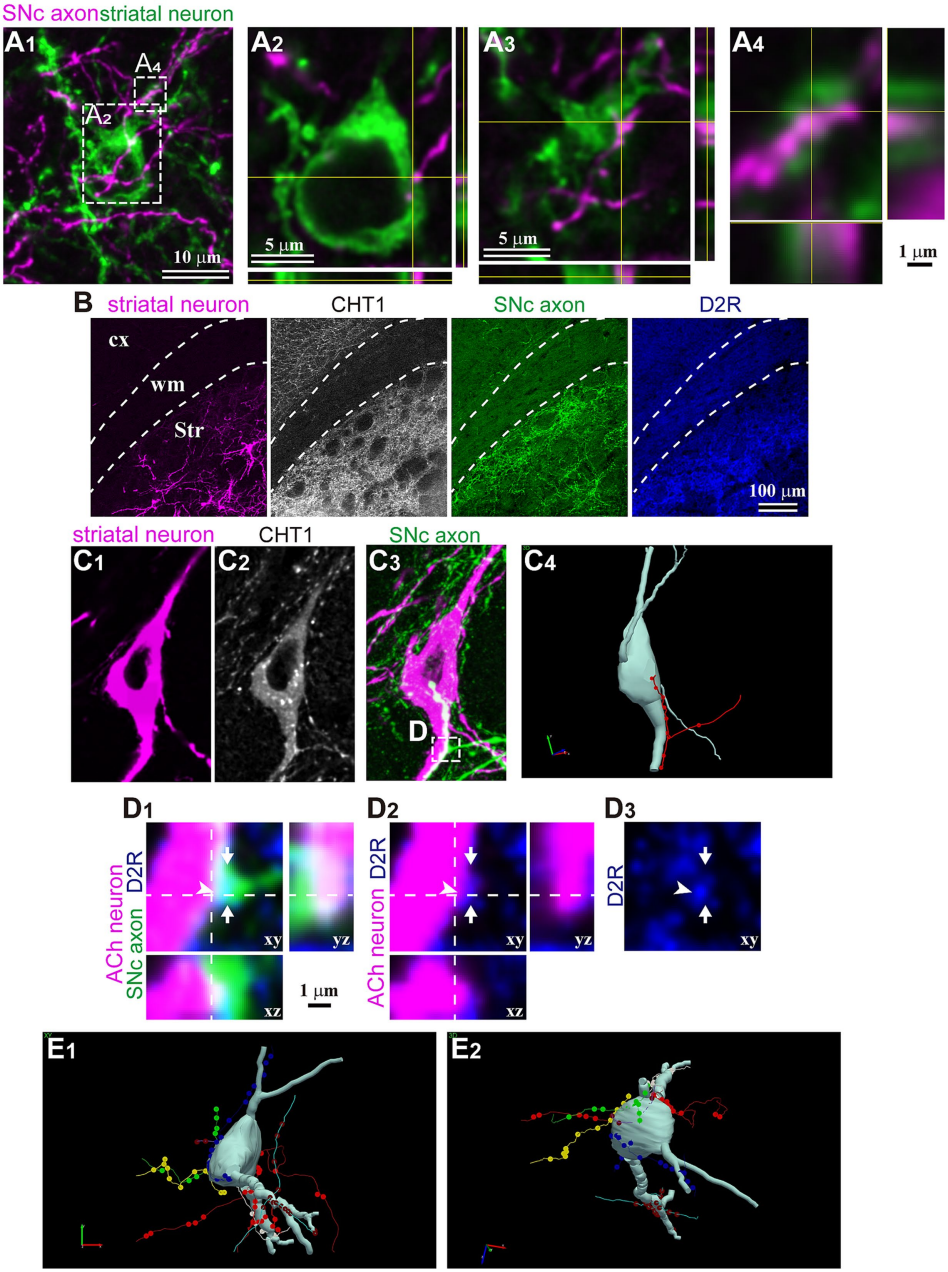
Magnified views of trans-neuronal labeled striatal neurons. **(A)** Confocal images of an aspiny striatal neuron. No tested maker was detected in the neuron. **(A1)** The SNc axon (magenta) and GFP-labeled cell body and dendrites. Planes of xy, xz, and yz are shown. **(A2–A4)** Magnified view of rectangle area shown in **(A1)**. **(A2)** One close apposition was found on the cell body. **(A3)** One of two close appositions on the proximal dendrite. **(A4)** Other appositions on the same dendrite than those in **(A3)** on the same dendrite. **(B)** Immunostaining against CHT1 and D2R to identify relationship between SNc axons and striatal neurons visualized using AAV1. **(C)** An example of cholinergic neurons and appositions close to the soma. A single axon segment formed 10 appositions in line. **(C1)** In this mouse, striatal neurons were visualized using AAV-Flex-mCherry. **(C2)** The neuron expressed CHT1, indicating its cholinergic (ACh) nature. **(C3)** The SNc axons were labeled with AAV-Flex-GFP. **(C4)** A rotated view of the 3D reconstruction of the cholinergic neuron (pale gray). An axon segment forming close appositions are shown in red. **(D)** Magnified confocal images of the rectangle area shown in **(C)**. The section was immunostained against dopamine receptor type 2 (D2R; blue). An axonal varicosity (arrow) formed a close apposition (arrowhead), close to which D2R was detected. **(E)** A 3D reconstruction of another cholinergic neuron. Six axon segments (represented in different colors) formed close appositions on the cell body and the proximal dendrites. Views from two angles are represented **(E1,E2)**. cx, cerebral cortex; Str, striatum; wm, white matter.

Finally, in a subset of fluorescent-labeled neurons, we directly examined the relationship between single DA axons and the labeled neurons. Using confocal laser scanning microscope (CLSM) images, we meticulously traced and reconstructed the labeled cell bodies and their dendrites (Figure 6). We then scrutinized for putative appositions (PAP) between DA axons and these striatal neurons. An instance of non-spiny neurons is shown in Figure 6A. This neuron did not express ChAT, and its cell body was small. We discerned PAPs on the cell body and along the proximal dendrite. For ChAT neurons, PAPs were frequently observed (Figures 6B–D; *N* = 8/8 ChAT neurons). DA axons also formed PAPs onto PV neurons (*N* = 2/2). Occasionally, single consecutive axon segments formed multiple PAPs on the cell body and/or proximal dendrites. In certain instances, the expression of dopamine receptor type 2 (D2R) was found in close proximity to PAPs, suggesting the presence of functional DA synapses (Figure 6D).

## 4 Discussion

In the present paper, we demonstrated an example of applying AAV1-driven anterograde trans-neuronal labeling to the DA neural circuitry. However, it is important to note, as discussed below, that in the case of DA neurons, we could not definitively rule out the possibility that AAV1 transportation occurred exclusively at synaptic sites. Consequently, we refrain from using the term “trans-synaptic” when referring to DA neurons in this context. We delve into methodological considerations regarding the application of AAV1 to DA neurons, highlighting its potential advantages in unraveling the complexities of the DA neural circuitry. In addition, the differences observed among striatal neuron types may reflect different mechanisms of DA transmission within the striatal interneurons.

### 4.1 Methodological consideration

Zingg et al. (2017, 2020), the pioneers in AAV1 trans-synaptic labeling, suggested that applying this method to the DA system might not be suitable due to the extensive bidirectional connections involved. In this study, we demonstrated that AAV1 injection into the SNc allows for the labeling of striatal interneurons, which can be analyzed as an instance of anterograde trans-neuronal transportation of AAV1. However, it is still crucial to exercise caution, as discussed below.

Previous reports have indicated that AAV1 can be retrogradely transported to cell bodies through their axons projecting to the injection site. When examining the SNc-striatum projection, retrograde labeling can occur in dMSNs that project to the SNr and/or the SNc, as previously stated by Zingg et al. (2020). Because it is challenging to distinguish between anterograde trans-neuronal transport and retrograde transport, we focused primarily on striatal interneurons. The proportion of labeled MSNs in total labeled neurons (61%) (Figure 5C) was lower than the proportion of MSNs in total striatal neurons (around 95% in rodents) (Graveland and Difiglia, 1985). This suggests that the labeling more frequently occurred toward interneurons through anterograde trans-neuronal transport, and that retrograde labeling of MSNs might not strongly occur. Interestingly, the proportion of Cre-expressing neurons was not higher in Ctip2-expressing neurons, namely MSNs (Table 3), than in the total population of interneurons. If retrograde labeling occurred frequently, the proportion would increase beyond that of interneurons, however, the result was the opposite. Thus, this observation implies that even though the Cre-expression of MSNs can be derived from a mixture of anterograde trans-neuronal and retrograde labeling, anterograde trans-neuronal labeling may chiefly occur. However, we cannot exclude the possibility that MSNs were not labeled by the transneuronal process, but rather, by retrograde transport. Labeling of iMSNs using AAV1, which do not project to SN, can be considered as anterograde trans-neuronal transportation, akin to the case of interneurons.

In addition, ChAT, PV, and NOS neurons were labeled more than expected from observed cell type composition in intact mice, while SOM neurons were fewer (Figures 4C, 5C; Tables 2, 3). Regarding the slight differences among NOS, SOM, and NPY neurons, it is likely to be contradictory because these three markers express largely in the same population of neurons. Certainly, the smaller populations of neurons express either of them, which can affect the results. Alternatively, the differences can occur just by chance. Notably, the proportion of interneuron types that was observed in this study in the dorsolateral striatum of intact mice was not largely divergent (Figure 4C; Table 2; Kawaguchi et al., 1995; Munoz-Manchado et al., 2016). This suggests that differentiation in neural communication between DA neurons and each type of striatal interneurons may influence AAV1 transportation. However, we observed a slight difference in the cell type composition between Cre expression and GFP/RFP expression (Figures 4C, 5C). Several possibilities can be considered to explain these observations. First, since the locations of AAV1 injections into the SNc were not consistently identical among mice, DA neurons infected by AAV1 could differ in terms of their location within the SNc. It is known that the distribution of SNc DA neurons is related to their neuron types (Menegas et al., 2015; Poulin et al., 2018, 2020; Luppi et al., 2021), potentially leading to differences in the labeled neurons within the striatum. In this scenario, a specific relationship between DA neuron types and striatal interneuron types may be at play. Second, building on the first point, DA neurons encompass groups of neurons that co-transmit glutamate and/or GABA alongside DA (González-Hernández et al., 2001; Yamaguchi et al., 2013; Poulin et al., 2018). It is conceivable that AAV1 particles can only pass through regions where glutamatergic or GABAergic synapses are present, even in the case of DA neurons. Third, it is worth considering that the sensitivity of the methods used to detect AAV1 transportation could affect the results. Cre-expression was detected using immunofluorescence in Figure 4, whereas fluorescent expression in the striatal neurons was driven by a Cre-loxP system (Figure 5). This disparity in detection methods may contribute to the observed differences in Cre expression among interneuron types. In another aspect, the CV of Cre-expression among ROIs was higher in ChAT, PV, and NOS neurons (Table 3). The potential innervation bias of SNc DA neurons may relate to these differences. Conversely, the CV for Cre-expression among ROIs was low in MSNs (Table 3), suggesting MSNs can be labeled equally irrespective of the location in the striatum. However, we cannot definitively conclude whether this occurred purely by chance, or if there are cell-type-dependent differences play.

Our study revealed that nearly all axon segments expressed DAT, indicating that they originated from DA neurons, and the level of contamination from other neurons was almost neglectable. With regard to expression of VGluT2 or VGAT in AAV1-labeled axon varicosities, previous research has indicated that the co-expression of DA and glutamate or GABA in a small proportion of DA neurons (González-Hernández et al., 2001; Yamaguchi et al., 2013; Morales and Root, 2014; Trudeau et al., 2014; Zhang et al., 2015). Chuhma et al. (2018) also reported the actual co-transmission of glutamate and DA in the dorsolateral striatum onto cholinergic neurons. In the case of GABA co-transmission, it has been shown that unlike pure GABAergic neurons, DA neurons do not use VGAT, instead, they utilize vesicular monoamine transporter (VMAT) and GABA transporter (GAT1), to package GABA in the vesicles (Tritsch et al., 2012, 2014, 2016; Melani and Tritsch, 2022). Thus, with regard to the VGAT expression observed in this study, the detection may have been influenced by the resolution limitation of our observation using CLSM, especially along the Z-axis. As shown in Figure 3C, the varicosities expressing VGluT2 or VGAT were not consistently located within a given axon segment, indicating that the labeled axons are not purely glutamatergic or GABAergic in nature. This is consistent with our observation of DAT expression in the labeled axons.

### 4.2 Possible advantages of AAV1-dependent tracing for dopaminergic neural circuits

The concrete confirmation of the actual presence of synapses between pre- and post-structures necessitates the use of electron microscopy. As elucidated by Matsuda et al. (2009), axon collaterals originating from a single DA neuron occupy a large spatial volume in the striatum and possess extremely dense varicosities. Consequently, systematic elucidation of the rules governing DA innervation onto striatal neurons at the single-cell level can be quite challenging. The potential advantage of AAV1 labeling lies in its simplicity: it allows visualization of pre- and post-synaptic neurons simultaneously, as demonstrated in this study. It holds the promise of investigating the presence or absence of synaptic structures between labeled SNc axons and labeled striatal neurons and can lead to the recognition of the mode of DA transmission, namely, synaptic or volume transmission. Such observations will be invaluable for understanding the mechanism underlying synapse formation by DA axons and its relationship with different types of striatal neurons. Moreover, confirmation of the presence or absence of synapses in the case of DA neurons can shed light on the machinery of AAV1 particle transportation. We speculate that if the machinery for volume transmission also facilitated the trans-neuronal transportation of AAV1 particles, the numerous varicosities along DA axons would likely lead to the labeling of a considerable number of striatal neurons than what we observed in this study (Figures 1D,E).

### 4.3 Does a given dopaminergic neuron deeply relate to some interneurons selectively?

We demonstrated AAV1-driven labeling of striatal interneurons, and some of these interneurons exhibited close appositions with DA axon varicosities on the proximal dendrites and/or cell bodies (Figure 6). This suggests that AAV1 particles released in proximity to the cell body or thick dendrites may more efficiently label neurons. In the case of glutamatergic or GABAergic synapses, larger postsynaptic structures, like those on thick dendrites or cell bodies, have larger synaptic areas (Holderith et al., 2012; Kubota et al., 2015, 2016). If DA synapses follow a similar pattern, it could imply that neurons with appositions close to the soma are more likely to be labeled efficiently. However, it is important to note that MSNs, in which only distal appositions were frequently found, were also labeled. Thus, the precise relationship between the location of the appositions and the efficiency of labeling must be clarified in the future study.

In certain instances, a single consecutive axon segment formed multiple appositions onto a single neuron (Figures 6C,D). Notably, DA receptors are not only expressed in MSNs but also interneurons, with the specific combination of DA receptor types varying depending on the interneuron type (for review, Kreitzer, 2009). This unique relationship suggests the possibility of selective connections between a particular DA neuron and striatal neurons. Traditionally, due to the extensive and dense axon collaterals of DA neurons and their characteristic mode of volume transmission, it has been thought that selective connections, as observed in neurons that utilize fast transmitters, are unlikely to form. Our present results suggest that at least some DA neurons can establish specific trans-neuronal networks, in which a limited number of DA neurons can strongly influence a given striatal neuron. Moreover, this kind of relationship may vary depending on the subregions of the striatum (Chuhma et al., 2023). Our observation may help to recognize the mechanism that restricts the spatial and temporal dimensions of DA function.

## 5 Concluding remarks

To summarize, this study showed that AAV1-driven trans-neuronal labeling can be applied to research on nigra-striatal projections of DA neurons. With careful consideration of contamination of retrograde labeling and the source of projection neurons, it is worthwhile to investigate cell-to-cell relationships between DA neurons and striatal neurons.

## Data availability statement

The original contributions presented in the study are included in the article/Supplementary material, further inquiries can be directed to the corresponding author.

## Ethics statement

The animal study was approved by the Animal Care and Use Committee of Hokkaido University. The study was conducted in accordance with the local legislation and institutional requirements.

## Author contributions

FK: Conceptualization, Data curation, Formal analysis, Funding acquisition, Investigation, Methodology, Project administration, Resources, Supervision, Validation, Visualization, Writing – original draft, Writing – review & editing. YY: Data curation, Formal analysis, Investigation, Visualization, Writing – original draft, Writing – review & editing. KK: Resources, Writing – review & editing. FF: Conceptualization, Funding acquisition, Investigation, Resources, Supervision, Writing – original draft, Writing – review & editing.

## Footnotes

^1^ http://www.r-project.org/
