# Supplementary figures and images for: Anterograde trans-neuronal labeling of striatal interneurons in relation to dopamine neurons in the substantia nigra pars compacta
Source: Front Neuroanat. 2024 Feb 28;18:1325368. doi: 10.3389/fnana.2024.1325368 (PMC10933013; doi:10.3389/fnana.2024.1325368)

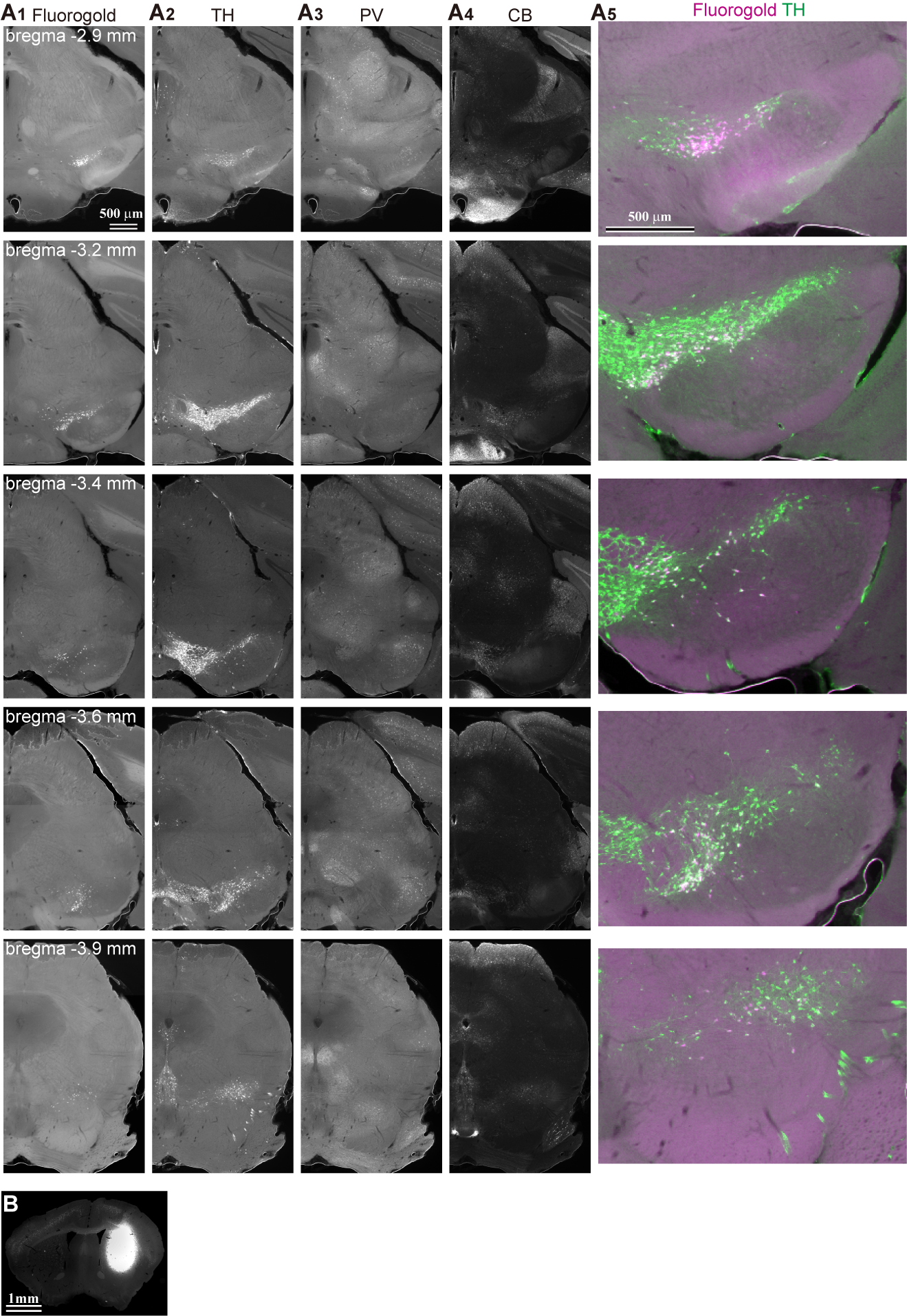

Supplement: Supplementary file 1 [file Image_1.tif]
